# The Specific α1-Adrenergic Receptor Antagonist Prazosin Influences the Urine Proteome

**DOI:** 10.1371/journal.pone.0164796

**Published:** 2016-10-25

**Authors:** Mindi Zhao, Jianqiang Wu, Youhe Gao

**Affiliations:** 1 Department of Pathophysiology, Institute of Basic Medical Sciences, Chinese Academy of Medical Sciences, School of Basic Medicine, Peking Union Medical College, Beijing, China; 2 Department of Biochemistry and Molecular Biology, Beijing Normal University, Gene Engineering and Biotechnology Beijing Key Laboratory, Beijing, China; Emory University Department of Medicine, UNITED STATES

## Abstract

Urine, reflecting many changes in the body, is a better source than blood for biomarker discovery. However, even under physiological conditions, the urine proteome often varies. Understanding how various regulating factors affect urine proteome helps link changes to urine proteome with urinary biomarkers of physiological conditions as well as corresponding diseases. To evaluate the possible impact of α1-adrenergic receptor on urine proteome, this study investigated effects of the specific inhibitor prazosin on the urine proteome in a rat model by using tandem mass tagging and two-dimensional liquid chromatography-tandem mass spectrometry. A total of 775 proteins were identified, approximately half of which were influenced by prazosin treatment, indicating that the sympathetic nervous system exerts a significant impact on urine proteome. Eight significantly changed proteins were previously annotated as urinary candidate biomarkers. Angiotensinogen, haptoglobin, and beta-2 microglobulin, which were reported to be associated with blood pressure, were validated via Western blot. Prazosin is widely used in clinical practice; thus, these protein changes should be considered when studying corresponding diseases such as hypertension, post-traumatic stress disorder and benign prostatic hyperplasia. The related physiological activities of α1-receptors, controlling blood pressure and fear response might significantly affect the urine proteome and warrant further biomarker studies.

## Introduction

Biomarkers are the measurable changes associated with a physiological or pathophysiological process [1]. As a better biomarker source than blood, urine reflect multiple changes in the body, and is affected by many factors such as physiological conditions, age, gender, hormones and diseases [2]. It is difficult to determine which factors induce complicated urine proteome changes. Elucidating how various regulating factors affect the urine proteome allows the association of changes to the urine proteome with urinary biomarkers of physiological conditions in addition to diseases and their corresponding mechanisms. The urine proteome can be affected by anesthetics [3], which suggested that urinary proteins might be regulated by the central nervous system; thus, changes in central nerve system are reflected in urine. The sympathetic nervous system is one of two important components of the autonomic nervous system. Changes resulting from sympathetic nervous system activation or inhibition are often present in many diseases. However, whether the sympathetic nervous system can regulate the urine proteome has not been studied.

A1-receptors represent attractive and tangible targets for therapeutic intervention and have been shown to mediate sympathetic nervous system activity [4]. Antagonism of α1 receptors results in smooth muscle relaxation and a decrease in total peripheral resistance [5]. Prazosin, a specific α1-adrenergic receptor antagonist, has been used alone or with other medications to treat cardiovascular diseases such as hypertension and congestive heart failure[6, 7]. More recently, prazosin was suggested as a treatment for post-traumatic stress disorder (PTSD), which is a common disease with a comparatively high morbidity rate and severe comorbidities. Prazosin has been demonstrated to be safe and effective for the treatment of nightmares and sleep disturbances associated with PTSD [8], which might be partly because α1-adrenergic receptors are involved in fear response behaviors as well as startle and sleep response in the brain, decreasing levels of norepinephrine in the central nervous system[9]. In the urinary system, studies have indicated that α1-adrenergic receptor antagonists play important roles in modulating sympathetic and parasympathetic activity of the urinary bladder[10]. These α-adrenergic blockers, especially those that are specific to α-1a, have been implicated and used in the treatment of lower urinary tract symptoms such as symptomatic benign prostatic hyperplasia and urinary dysfunction in women[10, 11]. The α-1a receptor is actively involved in these diseases; thus, studying the specific antagonist prazosin might help in understanding the effects of the α-1a receptor on the urine proteome and provide clues into the regulatory mechanism of the urine proteome.

A prazosin study in rats using liquid chromatography-mass spectrometry (LC-MS) platform was performed. An animal model was used to limit the number of confounding factors and enable observation of associated changes from fewer samples. Urinary protein identification was performed in six samples that before and after prazosin-treatment, and the protein changes were then validated in the remaining twelve samples.

## Materials and Methods

### Experimental rats

18 male Sprague-Dawley rats (12 weeks old) were purchased from the Institute of Laboratory Animal Science, Chinese Academy of Medical Science & Peking Union Medical College. The experiment was approved by the Institute of Basic Medical Sciences Animal Ethics Committee, Peking Union Medical College (Animal Welfare Assurance Number: ACUC-A02-2014-007). The study was performed according to guidelines developed by the Institutional Animal Care and Use Committee of Peking Union Medical College. All animals were maintained with free access to standard laboratory diet and water with a 12 h light–dark cycle under controlled indoor temperature (22 ± 1°C) and humidity (65–70%) conditions. Rats were sacrificed under deep sodium pentobarbital anesthesia after feeding on the last day and all efforts were made to minimize suffering.

### Administration of Prazosin

All rats were randomly divided into two groups: one group was administered daily prazosin at 10 mg/kg/day via oral gavage (9 rats); the other control group (9 rats) received treatment via gavage with a matching volume of 0.9% NaCl saline vehicle. Before the prazosin treatment and at the seventh day after prazosin treatment, all rats were individually placed in the metabolic cages for four hours. Then the four-hour urine samples were used for proteomics analysis. Blood samples were collected from each individual to monitor changes in low-density lipoprotein (LDL), high-density lipoprotein (HDL), triglycerides, cholesterol (Jiancheng Bioengineering Institute, China) and blood glucose (Roche, Germany). The experiment was conducted in two phases: for the discovery phase, differential urinary protein identification was performed in six independent samples that were collected respectively before and after prazosin treatment from three rats, and for the validation phase, samples were obtained from the twelve remaining samples (six before prazosin treatment and the other six after that treatment).

### Urine sample preparation and tandem mass tag (TMT) labeling

Urine was centrifuged at 2000 g for 30 min immediately after collection. Three volumes of acetone were added after removing the pellets, and the samples were precipitated at 4°C. Then, 8 M urea, 2 M thiourea, 25 mM dithiothreitol and 50 mM Tris were used to re-dissolve the pellets. The protein concentration of each sample was measured by the Bradford protein assay. The proteins were digested with trypsin (Promega, USA) using filter-aided sample preparation methods[12]. Briefly, 200 μg of protein were loaded on the 10kD filter unit (Pall, USA), and 200 μl UA (8 M urea in 0.1 M Tris–HCl, pH 8.5) was added to the filter unit and centrifuged at 14,000g for 40 min. Then 200 μl ABC (0.05M NH_4_HCO_3_ in water) was added and the centrifuged. 100 μl dithiothreitol solution (0.02 M dithiothreitol in ABC) was added to the filter unit and incubated for 1 hour. Centrifuge the filter units at 14,000g for 30 min. 100 μl iodoacetamide solution (0.05 M iodoacetamide in ABC) was added to filter unit and incubated in the dark for 40 min. Centrifuge the filter units at 14,000g for 30 min. Then the concentrate was dissolved in 50 mM NH_4_HCO_3_. Proteins were digested with trypsin (4 μg) at 37°C overnight. The digested peptides were desalted using Oasis HLB cartridges (Waters, USA).

The six urinary samples before and after the prazosin administration were individually labeled with 126, 127, 128, 129, 130 and 131 TMT reagents according to the manufacturer’s protocol (Thermo Fisher Scientific, Germany) and then analyzed with two dimensional (2D) LC/MS/MS.

### Reverse Phase Liquid Chromatography (RPLC)

All samples were fractionated using offline high-pH RPLC columns (XBridge, C18, 3.5 μm, 4.6 mm × 250 mm, Part No. 186003943; Waters, USA). The samples were loaded onto the column in buffer A1 (10 mM NH_4_FA in H_2_O, pH = 10). The elution gradient was 5–30% buffer B1 (10 mM NH_4_FA in 90% acetonitrile, pH = 10; flow rate = 1 mL/min) for 60 min. The eluted peptides were collected at one fraction per minute. After lyophilization, the 60 fractions were re-suspended in 0.1% formic acid and concatenated into 30 fractions by combining fractions 1 with 31, 2 with 32, and so on[13].

### LC-MS/MS analysis

Each fraction was analyzed three times using a reverse-phase C18 (3 μm, Dr. Maisch, Germany) self-packed capillary LC column (75 μm × 120 mm). The eluted gradient was 5%–30% buffer B (0.1% formic acid in acetonitrile; flow rate 0.3μl/min) for 60 min. The peptides were analyzed using a linear trap quadrupole (LTQ)-Orbitrap Velos mass spectrometer (Thermo Fisher Scientific, Germany). The MS data were acquired in data-dependent acquisition mode using the following parameters: 300–2,000 m/z range with the resolution set to 60,000; dynamic exclusion was employed with a 60 sec window to prevent the repetitive selection of the same peptide. Full scans and higher energy collisionally activated dissociation (HCD) scans were acquired in Orbitrap. The normalized collision energy for HCD-MS2 experiments was set to 40%.

### Data processing

All MS/MS spectra were analyzed using the Mascot search engine (version 2.4.1, Matrix Science, UK), and proteins were identified by searching against the Swissprot_2014_07 databases (taxonomy: Rattus, containing 7,906 sequences). The parameters were set as follows: the carbamidomethylation of cysteines and TMT labeling were set as fixed modifications and two missed trypsin cleavage sites were allowed. The precursor mass tolerance was set to 10 ppm, and the fragment mass tolerance was set to 0.05 Da.

Mascot search results were filtered using the decoy database method in Scaffold (version 4.3.2, Proteome Software Inc., Portland, OR). Peptide identifications were accepted if they could be established at greater than 99.0% probability to achieve a false discovery rate (FDR) less than 1.0%. Protein identifications were accepted if they could be established at greater than 99.0% probability to achieve an FDR less than 1.0% and contained at least 2 identified peptides. Protein probabilities were assigned using the Protein Prophet algorithm[14]. Proteins that contained similar peptides and could not be differentiated based on MS/MS analysis alone were grouped to satisfy the principles of parsimony. Proteins sharing significant peptide evidence were grouped into clusters. Scaffold Q+ was used to quantitate TMT labeling quantification, peptide and protein identifications. Acquired intensities in the experiment were globally normalized across all runs. The statistical test utilized in Scaffold Q+ was permutation test. The calculation type was median, the reference type was average protein reference and the normalization between samples was set “ON”. The minimum dynamic range was set to 1%.

### Western Blot

A total of 20 μg of urinary proteins from each individual sample (six before prazosin administration and six after) were loaded onto a 10% SDS-PAGE and transferred to a polyvinylidene fluoride (PVDF) membrane (Whatman, UK). Membranes were incubated overnight at 4°C with primary antibodies against Angiotensinogen (AGT), Haptoglobin (HP), Legumain (LGMN) or Beta-2 microglobulin (B2M) (Abcam, USA). The membranes were then washed and incubated with peroxidase-conjugated anti-rabbit IgG (ZSGB-bio, China) at room temperature for 2 h, and the proteins were visualized using enhanced chemiluminescence (ECL) reagents (Thermo Fisher Scientific, USA). The ECL results were scanned and analyzed using an ImageQuant 400^TM^ Imager (GE Healthcare Life Sciences, USA) and the intensity of each protein band was quantified using Image J analysis software (National Institutes of Health, USA). The intensity of each protein before and after prazosin administration were analyzed by Mann-Whitney test. The unchanged protein “albumin” from MS results was used as loading control.

## Results and Discussion

### Characterization of prazosin-administered rats

**Fig 1:**
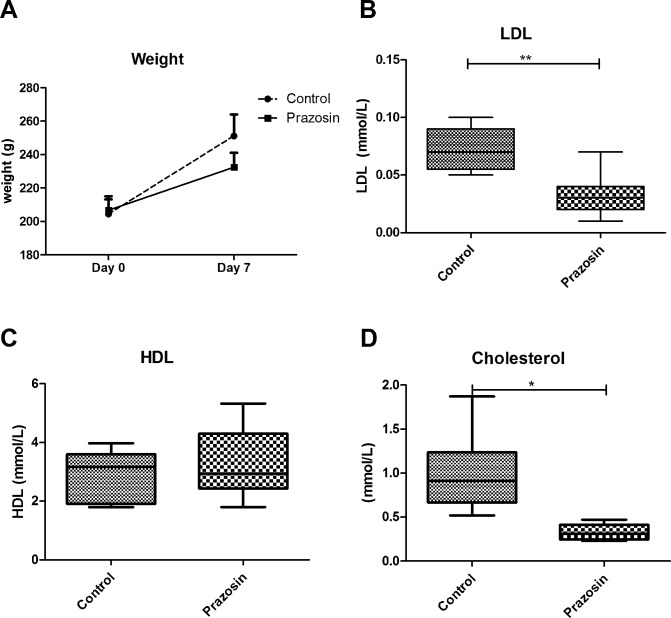
Effect of prazosin on rats measured at seven days. A: Body weights measured at the beginning and the end of the experiment in the control and experimental groups. B, C and D: Serum LDL, HDL and cholesterol measured at seven days in the control and prazosin-administered rats.

To investigate the effect of the specific α1-adrenergic receptor antagonist prazosin on the urine proteome, rats were treated separately with prazosin or a vehicle for seven days. The body weight was measured at regular intervals throughout the experiment. The mean body weight at day 7 was 232 g in the prazosin-administered rats and 251 g in the control rats (P value < 0.05). The ten rats in the prazosin-administered group exhibited a slower increase in body weight at day 7 than those in the control group (Fig 1A). Because the α adrenergic agonists were considered to stimulate energy metabolism[15], prazosin may have some direct or indirect effects on the body weight.

Studies have reported that the α1-adrenergic blockade was related to the serum lipid levels[16], therefore, the serum cholesterol, triglyceride, HDL and LDL levels were measured before and during the prazosin treatment. Consistent with previous studies, the serum LDL was significantly lower (p < 0.001) after seven days of prazosin treatment (Fig 1B). The mean concentration was 0.03 mmol/L, compared with 0.07 mmol/L for the control. However, significant differences were not observed in the serum HDL (2.91 mmol/L, compared with 2.88 mmol/L in the control) (Fig 1C), which may have been caused by the short-term prazosin treatment. A significant decrease was observed in the serum cholesterol (0.33 mmol/L, compared with 0.99 mmol/L in the control, P value < 0.05) (Fig 1D). The blood glucose, triglycerides and urine volume were similar between the two groups.

### Urine proteome changes

**Fig 2:**
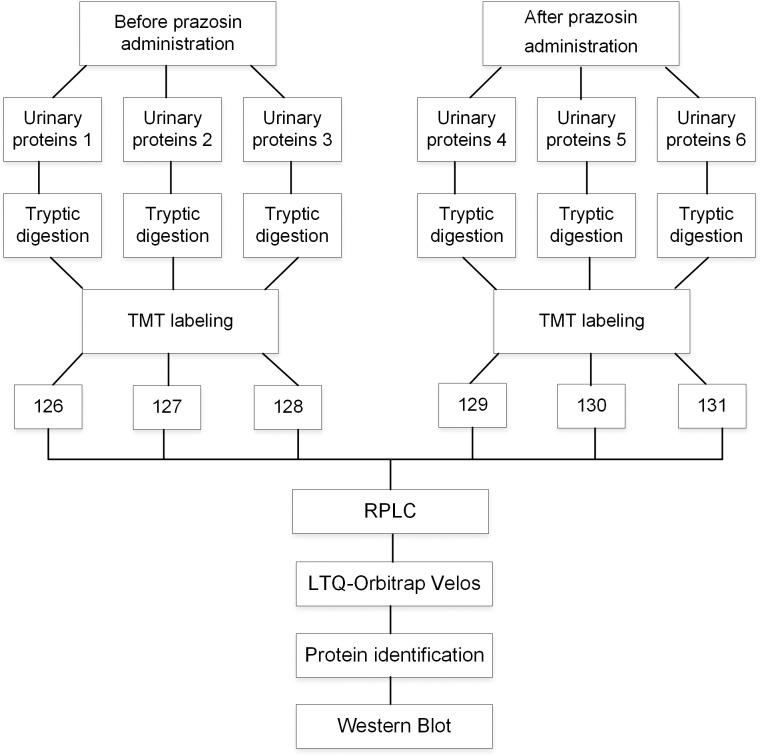
Workflow of the proteomic analysis. Urine samples were obtained before and after seven days prazosin administration. The labeled peptides were analyzed using 2DLC-MS/MS for identification and quantification.

In the discovery phase, urine samples collected from three pairs before and after seven days of prazosin treatment were obtained, respectively, for the TMT-2DLC-MS/MS analysis (Fig 2). The TMT-labeled samples were separated into 30 fractions, and each fraction was analyzed three times. All spectra were searched against the Swissprot database, and the resulting data were filtered using Scaffold software for protein identification and quantification. The identified peptides that are shared between two proteins were combined and reported as one protein group. A total of 54,591 spectra corresponding to 867 proteins with ≥ 2 peptides were identified with 1% FDR at the protein level were identified, among them, 775 proteins were quantified by TMT-labeling analysis. All identification and quantitation details are listed in S1 Table.

For all three pairs of samples, most of the proteins exhibited a similar response trend under prazosin administration according to the quantitative mass spectrometric analysis. Of the protein changes, 299 had a P value < 0.05 (statistical test utilized in Scaffold Q+ was a permutation test). Among these proteins, 10 up-regulated proteins showed a ≥ 1.50-fold change in each of the three samples that received prazosin, whereas 11 proteins demonstrated a decrease, with a fold change ≤ 0.67. Table 1 depicts the significant protein changes in the urine following the prazosin treatment.

**Table 1:** Details of changed urinary proteins identified in the TMT experiment.

ID	Protein Name	MW	Fold Change			Human ortholog	Biomarker use
			1	2	3		
P02761	Major urinary protein	21 kD	2.6	2.1	3.2	No	acute renal failure[17]
P06866	Haptoglobin	39 kD	2.9	3.4	2.3	Yes	diabetic nephropathy[18]
P07151	Beta-2-microglobulin	14 kD	1.5	2	1.8	Yes	acute tubular injury[19]
P01015	Angiotensinogen	52 kD	2.2	1.8	2.3	Yes	primary hypertension[20]
P10758	Lithostathine	19 kD	2.1	1.8	1.7	Yes	dents disease[19]
Q9R0J8	Legumain	49 kD	1.5	1.9	1.9	Yes	
B0BNN3	Carbonic anhydrase 1	28 kD	3.5	1.5	1.9	Yes	bladder cancer[21]
P26772	10 kDa heat shock protein, mitochondrial	11 kD	2	1.6	1.8	Yes	
P19223	Carboxypeptidase B	48 kD	2.1	2.2	2.3	Yes	
P19999	Mannose-binding protein A	25 kD	1.9	1.7	2.4	No	
P14046	Alpha-1-inhibitor 3	164 kD	0.5	0.6	0.6	Yes	
Q64319	Neutral and basic amino acid transport protein rBAT	79 kD	0.6	0.6	0.5	Yes	protein excretion with sodium loading[22]
P14668	Annexin A5	36 kD	0.5	0.6	0.5	Yes	acute renal failure[17]
Q9WTW7	Solute carrier family 23 member 1	65 kD	0.6	0.3	0.5	Yes	
Q06496	Sodium-dependent phosphate transport protein 2A	69 kD	0.5	0.6	0.4	Yes	
Q64602	Kynurenine/alpha-aminoadipate aminotransferase, mitochondrial	48 kD	0.6	0.5	0.5	Yes	
P70502	Solute carrier organic anion transporter family member 1A3	74 kD	0.6	0.6	0.5	Yes	
O35913	Cluster of Solute carrier organic anion transporter family member 1A4	73 kD	0.6	0.5	0.6	No	
O08839	Myc box-dependent-interacting protein 1	65 kD	0.6	0.6	0.5	Yes	
Q71MB6	Solute carrier organic anion transporter family member 4C1	79 kD	0.6	0.4	0.6	Yes	
A4KWA5	C-type lectin domain family 2 member D2	26 kD	0.6	0.6	0.5	Yes	

All significant changed proteins were searched against the Urinary Protein Biomarker Database[23]. A total of eight proteins had been annotated as urinary candidate biomarkers (Table 1). Of the 21 proteins that significantly changed (11 increased and 10 decreased), 18 have human orthologs. Using the Ingenuity Pathway Analysis (IPA) biomarker tool, four proteins had related annotations. Angiotensinogen (AGT), which is an essential component of the renin-angiotensin system, helps maintain the blood pressure balance and is frequently a major target for drugs that lower blood pressure[24]. Prazosin has been proposed to stimulate the renin-angiotensin system and increase serum angiotensinogen levels[25]. The plasma protein haptoglobin (Hp) has been reported to increase in response to inflammatory stimuli such as infection, injury or malignancy[26]. A previous study also confirmed a relationship between (Hp) phenotypes and blood pressure[27]. Beta-2 microglobulin (B2M) has been proposed as a prognostic factor for various diseases, such as mantle cell lymphoma[28] and acute kidney failure[19]. Similar to the previous two proteins of interest, significantly increased B2M excretion in urine has been observed in patients with severe blood hypertension[29]. All three of these proteins have been associated with blood pressure to some degree. Legumain (LGMN) is known to be positively expressed in many types of human tumors, including ovarian [30] and breast cancer[31], although whether LGMN is related to the effect of the α1-adrenergic receptor remains unknown. Disease-related emotional disturbances might affect the urine proteome via the α1-adrenergic receptor.

**Fig 3:**
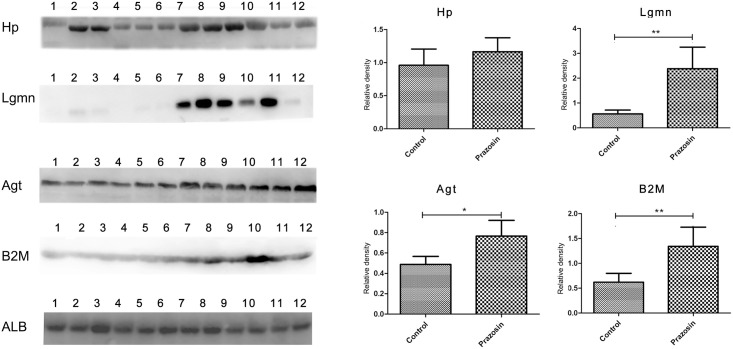
Western blot analysis of HP, LGMN, AGT and B2M in the urine samples before and after seven days of prazosin administration. The six samples on the left are before prazosin administration; the six samples on the right are after seven days of prazosin administration (* indicates a P value < 0.05, ** indicates a P value < 0.005).

Four changed proteins (HP, LGMN, AGT and B2M), which were increased in all three pairs, were selected for further validation in twelve additional individual urine samples via Western Blot, which was performed because of the relevance of these proteins to prazosin-related diseases as well as their relatively high abundance and commercially available antibodies. The expression of all four proteins was consistent with the MS results (Fig 3, the Mann-Whitney test was used to run the statistical analysis). In this study, elevated urinary AGT levels were associated with the prazosin treatment (p value, 0.006; 1.5-fold change). Urinary B2M showed a significant elevation (P value, 0.004; 2.1-fold change). Similarly, the LGMN levels were also significantly increased in the prazosin-administered sample (P value, 0.004; 4.0-fold change). However, HP was not significantly elevated after prazosin administration, but presented an increasing tendency.

### Functional annotation

**Fig 4:**
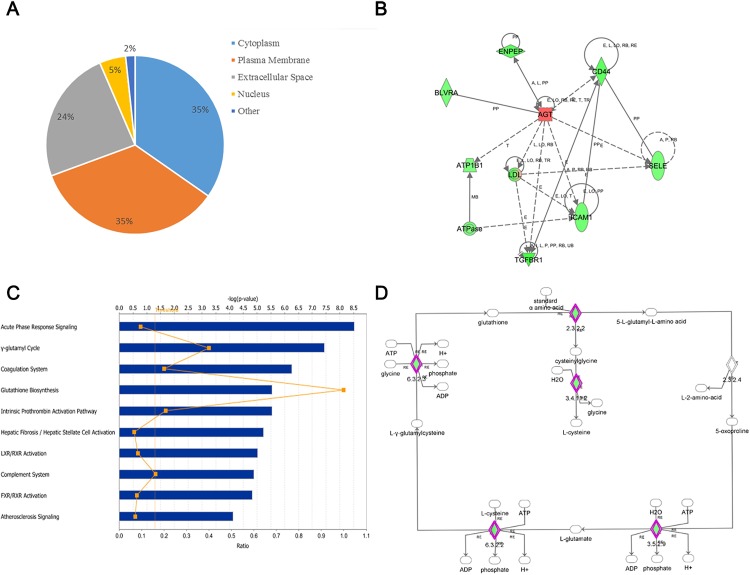
Functional analysis of the protein changes induced by the prazosin treatment. A) Subcellular location. B) AGT network. C) Top ten canonical pathways. The y axis shows the negative log of the p value. D) γ–glutamyl cycle pathway. The molecule marked in red is the protein identified in this study.

The 299 differentially expressed proteins were analyzed using the IPA tool to assess their association with important biological functions. These proteins were located in extracellular spaces (24%), the cytoplasm (35%) and the plasma membrane (35%) (Fig 4A). The ten canonical signaling pathways with the greatest changes are shown in Fig 4C. The canonical pathway analysis demonstrated that protein changes were highly associated with acute phase response signaling, the γ–glutamyl cycle and the coagulation system. Two representative canonical pathways in the prazosin-treated groups are the γ–glutamyl cycle and glutathione biosynthesis, both of which are important in amino acid biosynthesis and ATP energy metabolism. Glutathione not only protects against nerve damage[32], but also can haltongoing cycles of oxidative stress[33]. As shown in Fig 4D, the core components of the γ–glutamyl cycle pathway were identified in the prazosin-treated group.

The protein changes induced by prazosin might provide insights for further examinations of other pathological changes. As described above, AGT is a core component of the renin-angiotensin system and might be a target of prazosin [25]. The signature protein of AGT was further characterized based on protein–protein interactions constructed using IPA (Fig 4B). The network was divided into three parts according to the functions of the included proteins. Glutamyl aminopeptidase (ENPEP) and ATPase, which are important in energy and glutathione metabolism, are closely related to AGT. The inflammation-related molecules in the network (selectin (SELE), CD44 and intercellular adhesion molecule 1 (ICAM 1)) might be responsible for acute-phase signaling. Another interaction relevant to prazosin was linked to LDL because serum LDL was significantly decreased after the prazosin treatment.

Half of the identified urinary proteins showed an expression change (P < 0.05); thus, prazosin-related diseases such as hypertension, PTSD and fear response might influence the urine proteome and warrant further biomarker studies. Compared with the effects of anesthetics on the nervous system on the urine proteome [3], the protein changes in this study were substantially different and shared only one protein change. Changes in the urine proteome may also be caused by medicines such as diuretics [34] and anticoagulants [35], which indicates that the regulation of urinary proteins is complicated. More complicated urine patterns might represent connections to a greater number of physiological and pathophysiological conditions. This important property of urine increases the likelihood that it is a high-resolution and high-sensitivity biomarker source. Insight into how the various regulating factors affect the urine proteome can be used to associate changes in the urine proteome with urinary biomarkers of physiological conditions and diseases as well as their corresponding mechanisms.

## Supporting Information

S1 Table The identification and quantitation details. (XLSX)
